# Food and waterborne parasites in Africa - threats and opportunities

**DOI:** 10.1016/j.fawpar.2020.e00093

**Published:** 2020-10-20

**Authors:** Lucy J. Robertson, Simbarashe Chitanga, Samson Mukaratirwa

**Affiliations:** a Parasitology, Department of Paraclinical Science, Faculty of Veterinary Medicine, Norwegian University of Life Sciences, Adamstuen Campus, Ullevålsveien 72, 0454 Oslo, Norway; b Department of Biomedical Sciences, School of Health Sciences, University of Zambia, Zambia; c Department of Pathobiology, School of Veterinary Medicine, University of Namibia, Windhoek, Namibia; d School of Life Sciences, University of KwaZulu-Natal, Westville Campus, Durban, South Africa; e One Health Center for Zoonoses and Tropical Veterinary Medicine, Ross University School of Veterinary Medicine, Basseterre, Saint Kitts and Nevis

**Keywords:** Control, Knowledge, One Health, Sustainable Development Goal, Transmission, Water

## Abstract

This Special Issue (SI) was conceptualized on the basis that success in tackling foodborne and waterborne parasites (FBP and WBP) will contribute to achievement of seven of the United Nation's Sustainable Development Goals (SDGs). We chose to take a closer look at research on FBP and WBP in Africa, given that attaining these SDGs may be particularly challenging there.

In this SI we present 7 articles that provide particular insights into FBP and WBP from different regions in Africa. The articles take different approaches. Three papers are reviews addressing “occurrence” (either widely, in terms of parasite and/or geography, or with focus on a specific parasite genus at a more regional level); all 3 articles emphasise the importance of a “One Health” approach regarding control and prevention of FBP and WBP, and the need for further research to fulfil the information gaps identified. Two articles then report on investigations regarding the knowledge and understanding of different communities in Africa regarding various FBP and WBP. These articles highlight lack of awareness among communities at risk, and also, perhaps of greater relevance, gaps in the knowledge of health workers regarding some FBP and WBP of public health importance. The final two articles are research articles regarding prevalence and occurrence of specific WBP, both as infections and in the environment.

This SI, while limited in depth and scope, provides insights into some of the current challenges associated with FBP and WBP in Africa that might result in a lack of success regarding attainment of the previously mentioned seven SDGs. We anticipate significant advances in research on FBP and WBP in Africa, and hope that a future SI on the same topic may present a more positive picture regarding the current status and research achievements.

## Highlights

• A Special Issue focussing on foodborne and waterborne parasites in Africa
• Different aspects from various regions and countries explored in a series of articles
• Highlights the importance of a One Health approach.
• Tackling these parasites is essential for achieving UN Sustainable Development Goals.

## Graphical abstract

**Figure:**
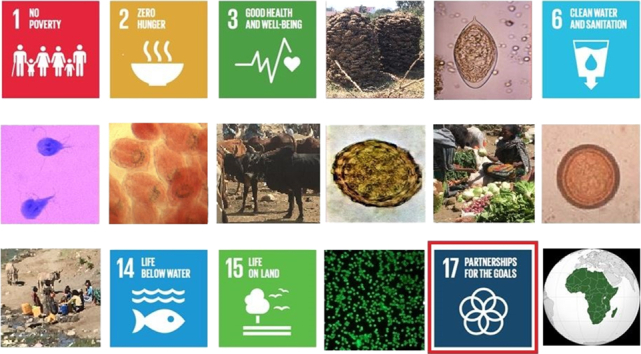

Africa is a vast continent, and any attempt to summarise particular characteristics of its land area of over 30 million km^2^, covering myriad climate areas and ecological zones, and with over 50 countries and more than 15% of the world's population, is doomed to failure. With this background, the intention of this Special Issue (SI), on foodborne and waterborne parasites (FBP and WBP) in Africa, was to create an SI that provides an opportunity for studies and reviews on FBP and WBP in Africa to take centre stage. Thus, the purpose of this SI was for researchers in Africa to deliver context for investigations and also to demonstrate that they, sometimes in collaboration with colleagues located elsewhere, are aware of the threats posed by FBP and WBP and are taking active steps to understand and mitigate against them.

The idea for this SI was partly instigated following discussion around the Sustainable Development Goals (SDGs) of the United Nations (UN), which were adopted by all UN Member States in 2015. These SDGs are hoped to provide a roadmap towards a peaceful and prosperous planet, both now and in the future, as part of the 2030 Agenda for Sustainable Development (https://sustainabledevelopment.un.org/sdgs). Among the 17 SDG, at least 7 (#1, #2, #3, #6, #13, #14, #15) are linked directly to threats and outcomes that may be associated with food and water, which are potential conduits for parasite transmission, whereas others have a more oblique association with FBP or WBP (e.g., SDG#4 “Quality Education” – it is well established that a child with worms may be unable to reach full educational potential). As we are now just a decade from 2030, it seems timely to revisit the SDGs in terms of FBP and WBP. However, creating a SI on the basis of the SDG and their associations with FBP and WBP, although an interesting concept, seemed both too ambitious and too nebulous, and we chose to focus specifically on Africa, a continent where many of these SDG seem particularly challenging to achieve. According to the 2030 Agenda for Sustainable Development, achievement of these SDG, and the 169 targets contained within them, should be based on multilateral partnerships and are a call for action among all countries, both developed and developing (as also encompassed in SDG#17 “Partnerships for the Goals”). Thus, it seemed appropriate to include in this SI, some articles that were not purely of African authorship but some that involved collaboration with partners from elsewhere.

Although natural resources are plentiful in Africa, it presents a particular challenge regarding achievement of the SDGs. It is the least wealthy region per capita and, using World Bank definitions, of the 31 countries globally classified as being in the lowest income group, 24 (77%) are in Africa (https://datahelpdesk.worldbank.org/knowledgebase/articles/906519-world-bank-country-and-lending-groups), with most African countries classified as having low-income or lower-middle income economies. Exceptions are Algeria, Botswana, Equatorial Guinea, Gabon, Libya, Mauritius, Namibia, and South Africa, which are classified as upper-middle income, and Seychelles classified as high income. Although Africa has a substantial biodiversity (being particularly known for having the greatest number of megafauna species), it is also beset by environmental issues, which include deforestation, desertification, water shortages, and poorly regulated urbanisation. In view of this, Africa has been reported to be the most vulnerable continent to climate change by the UN Intergovernmental Panel on Climate Change, and also with a low capacity for adapting to the negative effects of climate variability (Boko et al., 2007; Niang et al., 2014).

The 7 articles in this SI – originating from 7 different countries in Africa (Ethiopia, Mozambique, Nigeria, South Africa, Tanzania, Zambia, Zimbabwe; see Fig. 1), are divided into 3 overarching groups. The first group consists of three review articles that address occurrence, two of which overlap slightly; the one providing an African perspective of protozoan parasites that may be transmitted via food or water (Siwila et al., 2020; this issue) and the other a scoping review of waterborne parasitic infections in East Africa (Ngowi, 2020; this issue). The first article, which concentrates on protozoan FBP and WBP (particularly *Cryptosporidium* spp., *Giardia duodenalis*, *Cyclospora cayetanensis*, and *Entamoeba* spp.) concludes that with population growth in a situation of poorly maintained infrastructure, inadequate potable water supply, and often a lack of appropriate sanitary facilities, these parasites will continue to pose a threat to human health, and recommends that an integrated One Health approach is essential for their management, prevention, and control (Siwila et al., 2020). Supporting these data, the second article looks only at waterborne infections, but is not limited to protozoa, including also trematodes and nematodes, reporting that the majority of investigations have been on schistosomiasis. Other frequently reported waterborne parasitic diseases were giardiasis, soil-transmitted helminthiases, and amoebiasis, while cryptosporidiosis, isosporiasis, and drancunculiasis were reported less frequently. The author notes that many of the records of these infections are in the grey literature, such as hospital records, and thus should be included in any review that intends to obtain an overview of the disease burden caused by these parasites (Ngowi, 2020). Of particular interest is the paucity of reporting on waterborne cryptosporidiosis, given that it is considered a predominant waterborne disease in other reports, and seems potentially to contradict the message from Siwila et al. (2020). However, this probably reflects the difference in search (disease versus parasite), as well as the geographical restriction to Eastern Africa in the investigation described in this issue by Ngowi (2020). Indeed, Siwila et al. (2020) noted in their review in this issue, that most articles originated from North Africa, and the least from East Africa, indicating the same regional bias, although the reasons behind this are not clear.

**Fig. 1:**
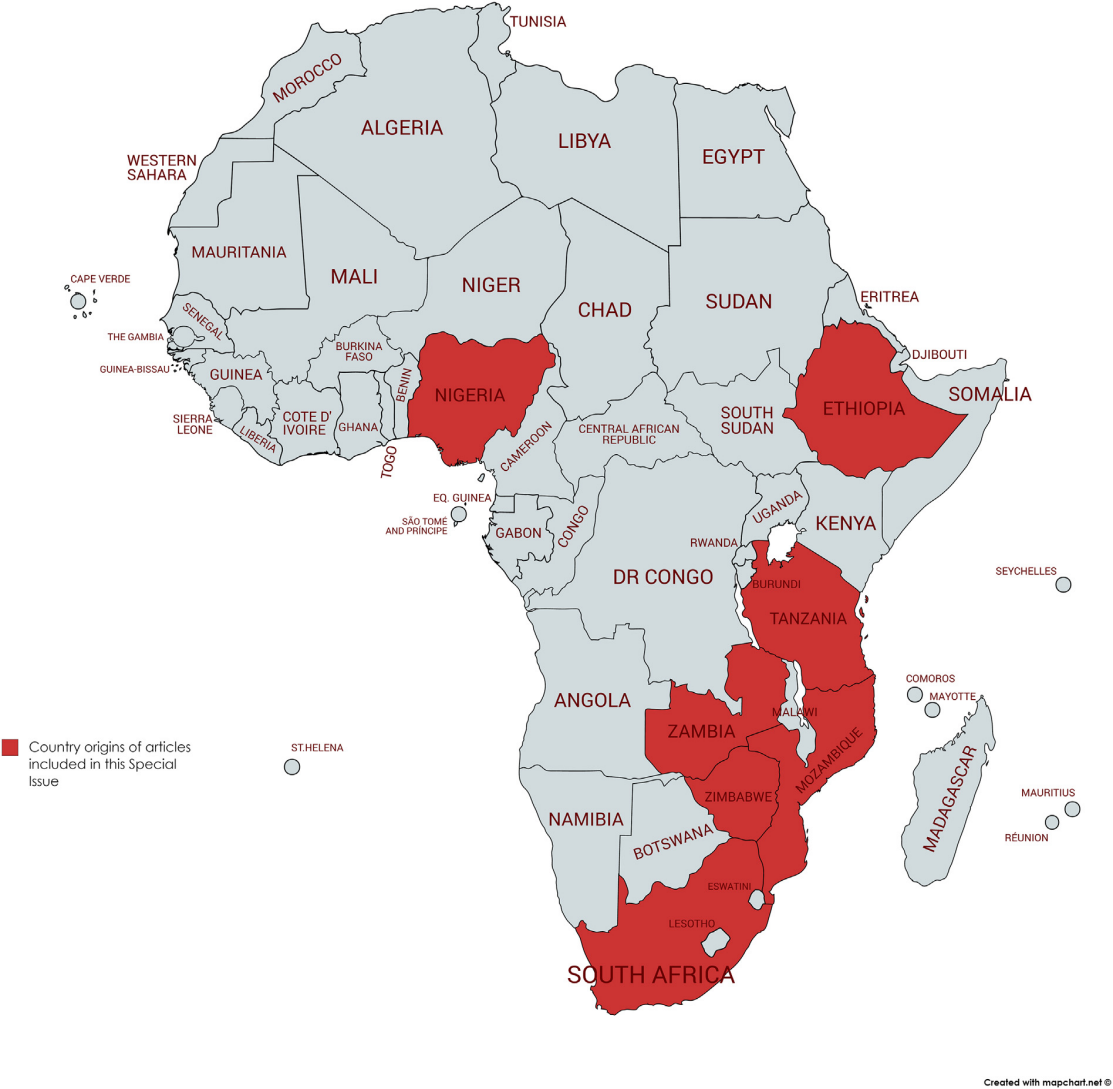
Countries from where Special Issue articles originated.

In addition, a systematic review in this issue by Miambo et al. (2020) considered echinococcosis in people and animals in Southern Africa Development Community (SADC) countries. The authors concluded that the paucity of epidemiological information regarding this important (potentially food and waterborne) parasitic zoonosis among these 15 SADC countries, particularly regarding gaps in prevalence data for both humans and animals, is indicative of a lack of investment at the governmental level throughout the region, and emphasised the need for prospective studies. In particular, the authors note that they were unable to find any reports/records of *Echinococcus* or echinococcosis from either Malawi or Lesotho, although both countries share borders with other countries that are endemic for this parasite. The authors suggest that this lack of reports or information, far from indicating an absence of occurrence of the disease in the two countries, more likely reflects that this parasitosis, despite being known to have a substantial health burden, is not considered a priority topic; they recommend an increase in One Health-based epidemiological studies to address this and other data gaps (Miambo et al., 2020; this issue).

Thus, these three articles provide overviews, both broad (WBP, foodborne/waterborne protozoan parasites) and focussed (echinococcosis) regarding the occurrence of these parasites from different African regional and continent-wide viewpoints. All articles advocate for the continuing need for targeted investment in research, control, and prevention following a “One Health” approach in order ascertain the true burden of these diseases in communities at risk. They also emphasise that the design and implementation of cost-effective strategies for their prevention and control should be a priority.

The second group of articles has people's knowledge and perceptions as the main thrust, with two articles, one from Nigeria and the other South Africa and Zimbabwe. These focus, respectively, on opinions and knowledge among Nigerian health workers regarding globally important FBP (Efunshile et al., 2020; this issue) and on community knowledge, perceptions, and social constructs regarding schistosomiasis in two separate study areas, one in South Africa and one in Zimbabwe (Mbereko et al., 2020; this issue). Of relevance is that further education on FBP for Nigerian healthcare professionals would be of value; of note is that only 35% of participants in the study were apparently aware that *Toxoplasma gondii* infection can be transmitted by consumption of undercooked meat, just 27% of participants considered fresh produce or water contaminated by positive human faeces could be transmission vehicles for *Taenia solium*, and little over 50% of respondents were apparently aware that diarrhoea was a symptom of cryptosporidiosis (Efunshile et al., 2020). Regarding schistosomiasis, the authors demonstrated that most participants in both study areas were aware of the disease and knew that it was waterborne, but there were differences regarding knowledge of the intermediate host (more knowledge at the South African study site) and the parts of the human body affected (more knowledge at the study site in Zimbabwe) (Mbereko et al., 2020). A lack of in-depth knowledge of the life cycle was identified, and the authors recommend that health education should be implemented, along with other strategies, for improving prevention and control of this important waterborne parasitosis (Mbereko et al., 2020).

The final two research articles address, respectively, asymptomatic *Giardia* infection in school children in Lusaka, Zambia (Tembo et al., 2020; this issue) and contamination of drinking water with *Cryptosporidium* and *Giardia* from different sources (protected or non-protected) in Tigray, Ethiopia (Kifleyohannes and Robertson, 2020; this issue). The work from Zambia indicated a prevalence of asymptomatic infections of around 10%, as detected by molecular analyses, indicating that anthroponotic transmission was predominant, although other likely vehicles of infection were not investigated (Tembo et al., 2020). The work from Ethiopia that focussed specifically on a potential transmission vehicle found that contamination of drinking water sources with *Cryptosporidium* oocysts was not extensive (5% of samples positive, all with low numbers of oocysts), and 16% of samples were contaminated with *Giardia* cysts, with numbers of cysts being as high as 22 cysts per 10 L (Kifleyohannes and Robertson, 2020). These papers, taken together, suggest that waterborne *Giardia* infection is a public health challenge in these African countries, and this might be the same elsewhere in Africa. Provision of adequately treated drinking water and protection of water sources from contamination with human sewage should be an area of focus (SDG #6). The report by Tembo et al. (2020) on a relatively high prevalence of asymptomatic infections highlights a potential source of contamination of the environment and, ultimately, infection of vulnerable members in the communities.

We are well aware that these articles are woefully inadequate at providing a thorough overview of the current status of FBP and WBP in Africa; in particular, we would have liked to have had more emphasis on *T. solium*, the most important FBP globally (FAO/WHO, 2014; Torgerson et al., 2015). Indeed, a comprehensive literature search on *T. solium* in Africa indicated that there is gross under-reporting (Braae et al., 2015), and having articles on the status of the parasite in endemic African countries in this SI would have contributed towards filling this data dearth. A One Health approach has been recommended as the most effective control strategy for many FBP and WBP parasites, particularly *T. solium* (Gabriël et al., 2017). However, in order to evaluate the effectiveness of control interventions, it is essential that reliable and comprehensive baseline data are already in place. Although our SI includes relevant information on the protozoa, particularly *Cryptosporidium* and *Giardia*, and also on the important trematodes in *Schistosoma* spp., other FBP/WBP of relevance to Africa were barely touched upon in this SI. These include helminths such as *Ascaris lumbricoides* (Karagiannis-Voules et al., 2015; Karshima, 2018), *Fasciola* spp. (Mas-Coma et al., 2019), *Paragonimus* spp. (Cumberlidge et al., 2018), *Taenia saginata* (Dermauw et al., 2018; Hendrickx et al., 2019; Saratsis et al., 2019), *Toxocara* spp. (Omonijo et al., 2019), *Trichinella* spp. (Mukaratirwa et al., 2013), and *Trichuris trichiura* (Karagiannis-Voules et al., 2015). It is perhaps of relevance that these parasites are more associated with insidious diseases, which, although having a fundamental effect on health and life quality (DALY and QALY measures), tend to be less frequently acute or dramatic. Based on the work in this SI that investigated the knowledge and perceptions regarding some FBP and WBP (Efunshile et al., 2020; Mbereko et al., 2020; both in this issue), it would seem likely that there will be even less knowledge regarding these other relevant FBP and WBP.

Although we are of the opinion that the information provided in this SI give some indication of the challenges regarding FBP and WBP in Africa that may hinder achievement of several of the SDGs, it does not paint the whole story. We hope to revisit this theme in the future and be able to develop a follow-up SI that showcases articles presenting not only more information that cover a broader spectrum of FBP and WBP in Africa, but also more success stories. These may be simply more baseline data on otherwise neglected pathogens (Robertson, 2018) or may report on outbreaks that have been investigated, associations that have been explored, or interventions that have been successful. However, to achieve our aims we are reliant on training and research collaboration, partnerships with various stakeholders, and maintaining not only a One Health approach, but also integrating diverse skillsets, including, for example, economics and engineering, along with more basic parasitology knowledge. Development of relevant research projects and, importantly, securing funding for them is also essential.

We remain positive, and, with the SDGs due to be achieved by 2030, leaving no one behind (https://www.un.org/sustainabledevelopment/sustainable-development-goals/), this could also be an appropriate timeframe for development of the next SI on FBP and WBP in Africa. We look forward to that opportunity.

## Declaration of competing interests

The authors declare that they have no known competing financial interests or personal relationships that could have appeared to influence the work reported in this paper.
